# Choriocarcinoma complicated with intra-abdominal and intrapleural hemorrhage in pregnancy – case report

**DOI:** 10.3389/fonc.2023.1198553

**Published:** 2023-08-17

**Authors:** Kamila Jaz, Cezary Miedziarek, Ewa Piasek, Artur Florek, Ewa Nowak-Markwitz, Mikołaj Piotr Zaborowski

**Affiliations:** ^1^ Department of Gynecology, Obstetrics and Gynecologic Oncology, Division of Gynecologic Oncology, Poznan University of Medical Sciences, Poznań, Poland; ^2^ First Department of Anaesthesiology and Intensive Therapy, Medical University of Lublin, Lublin, Poland; ^3^ Institute of Bioorganic Chemistry, Polish Academy of Sciences, Poznań, Poland

**Keywords:** choriocarcinoma, pregnancy, chemotherapy, hemorrhage, metastases

## Abstract

**Background:**

Choriocarcinoma is a rare neoplasm, exceptionally uncommon during an ongoing pregnancy. The disease often has a metastatic character, causing severe symptoms from various anatomic sites like the lungs, central nervous system, vagina, pelvis, or liver. Due to the condition’s rarity, evidence on how to treat the choriocarcinoma originating during pregnancy remains scarce.

**Case presentation:**

Here, we present a case of a patient who developed choriocarcinoma before the 29th week of gestation. The neoplasm had a metastatic character, resulting in hemorrhage complicated by a hypovolemic shock. The patient underwent an emergency cesarean section and several surgeries to stop the massive hemorrhage. The treatment of the choriocarcinoma included chemotherapy with methotrexate followed by an EMA-CO regimen. The patient had a complete response to the therapy. The neonate suffered from complications related to prematurity.

**Conclusion:**

Metastatic choriocarcinoma can be a diagnostic and therapeutic challenge during ongoing pregnancy. Treatment of the disease can be associated with severe complications, but a complete response to chemotherapy is possible with a favorable long-term prognosis.

## Introduction

Choriocarcinoma is a rare malignancy that occurs mainly in women between 20 and 50 years of age, with a mean incidence of 1 in 40 000 pregnant women (1–3). Etiologically, choriocarcinoma may derive from any pregnancy; however, it is most commonly associated with a molar pregnancy or a miscarriage (4). A complete or partial hydatidiform mole increases the risk of developing choriocarcinoma with the incidence of 3 in 1000 pregnancies (5). Less frequently, choriocarcinoma may also result from non-gestational tumors and may be a part of poorly differentiated germ cell tumors (6). The initial symptom of choriocarcinoma is abnormal uterine bleeding (2). Before the disease is symptomatic, the diagnosis can be established based on an abnormally high level of serum human chorionic gonadotropin (hCG). However, a choriocarcinoma has high metastatic potential, for which an affected patient can present with symptoms derived from distant metastases - hemoptysis, neurological symptoms, or uncontrolled internal bleeding (6). Symptoms related to metastases may emerge first before a precise diagnosis of choriocarcinoma. The common locations of metastases are the lungs (80%), brain (39%), vagina (30%), pelvis (20%), and liver (10%) (6, 7). Metastatic invasive trophoblastic disease during concomitant normal pregnancy is exceptionally uncommon, which occurs in 1 in 160 000 pregnancies. So far, only 19 cases of metastatic choriocarcinoma during pregnancy have been reported in literature (6). Here, we present a rare case study of choriocarcinoma in pregnancy which was complicated with massive intra-abdominal and intrapleural hemorrhage due to metastasis. Following an emergency cesarean section at 29 weeks of gestation, the patient was managed in the intensive care unit, underwent abdominal surgeries due to recurrent hemorrhage, and subsequently received chemotherapy.

## Case presentation

### Initial symptoms

In June 2022, a 25-year-old gravida 2 para 1, who carried an uncomplicated singleton pregnancy at 29 weeks of gestation, presented to the Clinical Hospital of the Medical University of Lublin and complained of pain in the right upper quadrant of the abdomen for one day, which did not resolve after administering non-steroidal anti-inflammatory drugs. Two months prior to the presentation, the patient had an episode of blurred vision that occurred abruptly, repeated infrequently, and resolved spontaneously after a few days. Otherwise, her first pregnancy was a normal pregnancy in 2014, and she had a natural childbirth. She denied a miscarriage. She had not suffered from any chronic illness or taken any medications.

### Admission to intensive care unit

**Figure 1:**
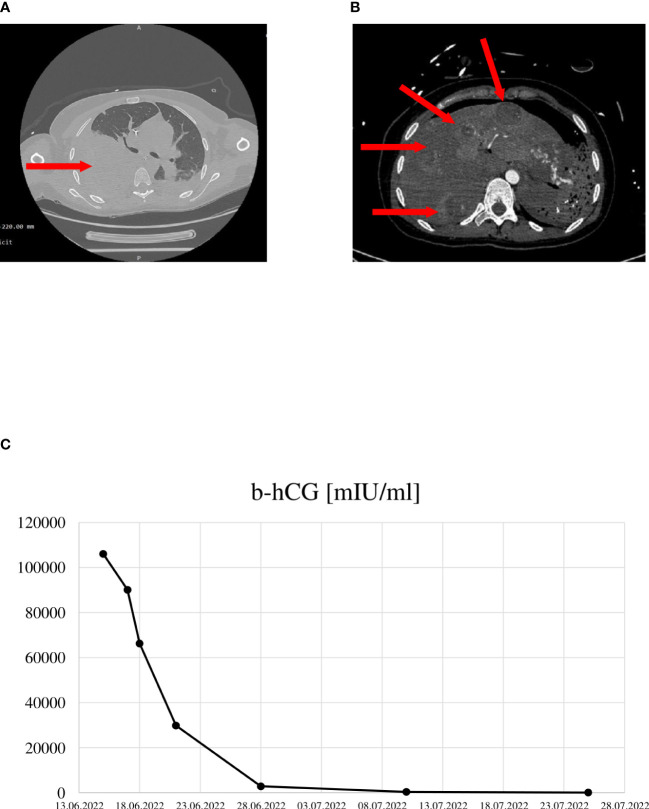
**(A)** Chest tomography imaging – hematoma of the right lung. **(B)** Abdominal tomography imaging - multiple vascular lesions in the lobes of the liver. **(C)** hCG concentration in the course of the disease.

The patient was admitted to the intensive care unit department (ICU) of the Clinical Hospital of the Medical University of Lublin due to massive internal bleeding. On the day of the admission, she presented with pain in her right upper abdomen and extensive sweating. She suddenly became unconscious and hemodynamically unstable due to a hypovolemic shock, requiring intubation, ventilation, aggressive fluid resuscitation and catecholamine administration. A computed tomography scan revealed that both pleural cavities were filled with heterogenous fluid. The fluid in her right pleural cavity was 60 mm in dorsal size, and its Hounsfield density was suggestive of a hematoma (**Figure 1A**). The imaging revealed numerous vascular lesions in the parenchyma of both lobes of the lungs. There were also numerous vascular lesions in her lungs and liver, a subcapsular hematoma within the hilum of the liver and free fluid in the peritoneal cavity (**Figure 1B**). The plasma concentration of hCG was 131 087 mlU/ml, which was abnormally higher than the normal level for her gestational age (8). The patient was transferred to the operating room.

### Surgical procedures and postoperative care

During the emergency laparotomy, a massive amount of blood and clots was removed. The surface of the uterus and the adnexa were macroscopically unchanged. A transverse cesarean section was performed, and a female neonate was delivered and transferred into the neonatal intensive care unit. The amniotic fluid was clear, with no signs of intrauterine bleeding. Macroscopically there were no signs of placental abruption. Due to massive intra-abdominal bleeding, a vertical midline incision of the abdominal wall was performed. A ruptured vascular lesion in the right lobe of the liver (segment 5), 9 cm in diameter, was visualized. Intense bleeding was observed in the region of the left lobe of the liver and spleen. A splenectomy was performed. Another 10 cm in diameter vascular lesion with intense bleeding was visualized in the left liver lobe. The liver packing was applied due to the persistent bleeding from the lesion in the left lobe and the ruptured region of the right lobe. The drainage was placed in the abdominal cavity. The blood loss during the surgery was approximately 2500 mL. The patient received 15 units of oxytocin administered intravenously. The placenta and the spleen were sent for a histopathological examination. The patient was transferred to the ICU department to continue cardiopulmonary support. On the following day, due to the persistent bleeding, the vascular lesions in the liver were embolized by an interventional radiologist. Two days later, the patient required another laparotomy because of recurrent intra-abdominal bleeding in the right lobe of the liver. Initially, the vessels were embolized; a biopsy was taken from the lesion. Six days after the initial surgery, the patient required the third laparotomy due to internal bleeding from the lesion in the right lobe of the liver – the lesion was sealed with a fibrin tissue glue. A drain was inserted into the right pleural cavity to evacuate the hematoma. As a result of the massive internal bleeding episodes, she required massive blood transfusion with a total of 20 units of packed red blood cells. On the eighth day of the initial laparotomy, the patient’s condition improved, and she was extubated.

### Chemotherapy

She was transferred to the Department of Gynecologic Oncology in Poznań, which is a centre of gestational trophoblastic neoplasia (GTN), for continuation of care. With the advanced disease and a high FIGO risk score of 14 (high-risk score ≥ 7) (9), a multi-agent chemotherapy using EMA-CO chemotherapy regimen (methotrexate, actinomycin D, etoposide, cyclophosphamide, and vincristine) was considered. However, we were concerned for her high risk of abrupt bleeding from the metastases in the liver and lungs, and, therefore, we adopted a stepwise approach by administrating methotrexate 300 mg iv. followed by the EMA-CO regimen after two days. After six days of chemotherapy, her hCG levels dropped from 106016 mIU/ml to 66264 mIU/ml, and the levels continued to drop (**Figure 1C**). The histopathological examination confirmed the diagnosis of choriocarcinoma with multiple metastases to the placenta, spleen, liver, and lungs. She developed several complications while receiving the chemotherapy. A hematoma recurred in her right pleural cavity, causing her a respiratory distress which warranted a drain insertion for three days, oxygen therapy and a transfusion of four units of packed red blood cells. After one week of the chemotherapy, she developed febrile neutropenia, which was treated with vancomycin. She also developed a cerebral venous sinus thrombosis, which was confirmed by a head magnetic resonance imaging (MRI). The radiology imaging excluded the presence of brain metastases. At that time, she revealed neurological signs and symptoms, such as impaired consciousness, visual hallucination, bradyphrenia and anisocoria. A neurologist’s opinion was sought, and heparin therapy and methylprednisolone were initiated. Otherwise, the MRI excluded brain metastasis. Her neurological symptoms improved after two days of anticoagulant therapy. She received a total of four cycles of the EMA-CO chemotherapy and achieved a complete remission. Since then, she remained under the ongoing care of the oncology clinic.

### Neonate

The newborn suffered from intraventricular bleeding related to prematurity, and she was managed by the insertion of a ventricular valve. She also required intensive rehabilitation for her partial hearing loss and increased muscle tone. Asymptomatic hemangiomas in the liver were detected in the newborn. However, the child was not directly affected by choriocarcinoma – hCG levels were within the normal range for a preterm neonate.

## Discussion

Choriocarcinoma is a rare malignant disease. It can be divided into gestational and non-gestational. The former originates from trophoblasts, whereas the latter originates from pluripotent germ cells (8, 9). Due to the frequent presence of paternal genetic material, gestational choriocarcinoma evokes a more intense immune response and is sensitive to chemotherapy (10). This disease subtype is characterized by high survival rates ranging from 91% to 100%, depending on the risk group (1). Non-gestational choriocarcinoma is derived from germ cells, has a much worse prognosis, and is less sensitive to chemotherapy (11). It is unrelated to pregnancy and was reported to have primary sites in the ovaries, lungs, cervix, endometrium, breast, bladder, and gastrointestinal tract (9). Due to the similarities in clinical and histological features, the differentiation between these two types can only be achieved by genetic testing which identifies the presence of paternal DNA in gestational choriocarcinoma (12). In the case of our patient, it remains unclear whether choriocarcinoma derived from her previous pregnancy. It is also hard to rule out that the patient might have had an early miscarriage that was interpreted as delayed menses.

It is challenging to establish the diagnosis of choriocarcinoma during an ongoing pregnancy. The general symptoms of malignancy can be improperly interpreted during gestation (13). Choriocarcinoma was considered in the differential diagnosis in our patient due to the high value of human chorionic gonadotropin (131 087 mlU/ml). It significantly exceeded the reference concentrations in 29 weeks of gestation, which, according to the Korevaar et al. study, usually is not higher than 74 719 mlU/ml after 25 weeks - hCG concentration during pregnancy typically reaches the maximum at the end of the first trimester and steadily decreases with the remaining gestation time (8). The complete disappearance of hCG was observed between 11 to 16 days (13) and 8 to 24 days (14). Cesarean section as a delivery method can influence the hCG concentration around the time of the procedure but does not impact the pace of its decrease (14).

Based on the clinical presentation of this patient, it was difficult to establish the diagnosis of choriocarcinoma during the ongoing pregnancy before the result of the histopathological examination was available. Bleeding from internal organs often occurs in metastatic disease (15, 16); however, it is not specific to choriocarcinoma. Moreover, women with choriocarcinoma during pregnancy, like in this case, do not demonstrate a typical clinical presentation of GTN, such as vaginal bleeding, uterine enlargement and the enlargement of bilateral ovaries or some of those can be attributed to pregnancy (17). Therefore, we could only rely on the disproportionately elevated hCG level and the multiple bleeding vascular lesions of her lungs and liver detected by the radiological imaging to support our clinical suspicion of a choriocarcinoma and initiate chemotherapy before the report of histopathological examination was available.

So far, only less than 20 cases of choriocarcinoma during pregnancy have been reported in the literature. Among them, there were three cases with pulmonary and liver metastases, which were also observed in our patient (6). Ding et al. described a pregnant patient with choriocarcinoma metastasizing to the vagina, lungs, and liver. The patient received two cycles of antenatal chemotherapy with EP regimen (etoposide and cisplatin), followed by a caesarean section at 31 weeks and 5 days of gestation and six cycles of EMA-CO regime postnatally. The patient responded well; the mother and child had no clinical symptoms in 4 years of observation. Although chemotherapy can be given during antenatal period, it was not feasible in our case because she had life-threatening internal bleeding, for which the surgical interventions were mandatorily required. In the case published by Kristiansen et al., the pregnant patient was diagnosed with choriocarcinoma metastasizing to the liver and the lungs. The patient underwent a cesarean section and hysterectomy at 22 + 3 weeks. The child died shortly after the procedure, and the mother was qualified for chemotherapy. One course of methotrexate and actinomycin-D was ineffective; therefore, the regimen was changed to BEP (Bleomycin, Etoposide, Cisplatin), which resulted in a complete response (18). Another case described by Brudie et al. presents metastatic choriocarcinoma in a pregnant patient with lung, liver, and brain lesions. The patient received four cycles of chemotherapy with the EMA-CO regimen before the planned induction at 32 weeks of gestation and continued the therapy afterward. The disease and treatment resulted in a persistent left-sided decrease in patients’ peripheral vision. There were no complications in the child (19).

It remains unclear whether an episode of blurred vision observed in our patient could be related to choriocarcinoma. The symptom could be caused by transient cerebral venous thrombosis (CVT). May et al. reported a case of a patient with choriocarcinoma and CVT. The authors indicate an increased risk of developing CVT in conditions predisposing to thrombosis, such as concomitant pregnancy and malignancy, as in the case of our patient (20).

Although the neurological signs and symptoms in our patient were attributable to a cerebral venous sinus thrombosis, it is important for clinicians to have an initial strong suspicion of brain metastasis (21–24), which happens in 39% of cases (6, 7). The imaging modalities of choice for the diagnosis of brain metastasis include CT and MRI. If the imaging tests are inconclusive, a serum: cerebrospinal fluid hCG concentration ratio below 60 can be used as an alternative method to diagnose brain metastasis (21, 25).

Avoiding massive bleeding from liver metastases during chemotherapy was a therapeutic challenge in managing our patient. According to Lurain et al., massive hmorrhage is the most common cause of death in patients with metastatic gestational choriocarcinoma (26). The highest risk of bleeding is at the beginning of chemotherapy due to the tumors high chemosensitivity resulting in rapid necrosis of the lesions (27). Instead of administering the standard EMA-CO regimen for our patient with a metastatic choriocarcinoma and a FIGO risk score of more than 7 (FIGO risk score = 14) (28), an initial single-agent chemotherapy with MTX before escalating it to EMA-CO regimen was decided upon. The rationale of this alternative approach was to reduce her risk of bleeding from the liver lesions by slowing down the rate of vascular necrosis. This similar strategy has also been described by Ding et al, in which a lower dose of chemotherapy with EP regimen (etoposide and cisplatin) was utilized in an antenatal mother to reduce the risk of massive bleeding from metastatic lesions, multiorgan failure and fetal complications (6). The emergency treatment option for massive bleeding from liver metastases is a selective temporal catheterization of the arteries supplying the metastases, which is considered a safer and more efficient method of bleeding control than packing or suturing (27, 28). Nevertheless, an arterial embolization does not completely eliminate the risk of recurrent bleeding. In our case, the multidisciplinary approach, which included surgical bleeding control, the correction of anemia and coagulopathy and assisted radiological embolization, has enabled her to achieve a fast recovery from the life-threatening condition, which rendered her eligible for the curative chemotherapy.

## Conclusion

Choriocarcinoma during normal pregnancy is extremely uncommon, and its clinical presentation may not be straightforward. If diagnosed, the disease is potentially curable; however, its complications during treatment can be severe and difficult to manage. Therefore, managing this type of cases requires multidisciplinary team management.

## Data availability statement

The original contributions presented in the study are included in the article/supplementary material. Further inquiries can be directed to the corresponding authors.

## Ethics statement

Written informed consent was obtained from the individual(s) for the publication of any potentially identifiable images or data included in this article.

## Author contributions

KJ, CM, AF, EN-M, and MPZ contributed to the conception and design of the study. KJ, CM, EP, and MPZ organized the data. KJ and CM wrote the first draft of the manuscript. KJ, CM, and MPZ wrote sections of the manuscript. All authors contributed to the article and approved the submitted version.
